# Revisiting GMOs: Are There Differences in European Consumers’ Acceptance and Valuation for Cisgenically vs Transgenically Bred Rice?

**DOI:** 10.1371/journal.pone.0126060

**Published:** 2015-05-14

**Authors:** Anne-Cécile Delwaide, Lawton L. Nalley, Bruce L. Dixon, Diana M. Danforth, Rodolfo M. Nayga, Ellen J. Van Loo, Wim Verbeke

**Affiliations:** 1 Department of Agricultural Economics and Agribusiness, University of Arkansas, Fayetteville, Arkansas, United States of America; 2 Department of Agricultural Economics, Ghent University, Ghent, Belgium; 3 Adjunct professor, Korea University and Norwegian Agricultural Economics Research Institute; University of Tsukuba, JAPAN

## Abstract

Both cisgenesis and transgenesis are plant breeding techniques that can be used to introduce new genes into plant genomes. However, transgenesis uses gene(s) from a non-plant organism or from a donor plant that is sexually incompatible with the recipient plant while cisgenesis involves the introduction of gene(s) from a crossable—sexually compatible—plant. Traditional breeding techniques could possibly achieve the same results as those from cisgenesis, but would require a much larger timeframe. Cisgenesis allows plant breeders to enhance an existing cultivar more quickly and with little to no genetic drag. The current regulation in the European Union (EU) on genetically modified organisms (GMOs) treats cisgenic plants the same as transgenic plants and both are mandatorily labeled as GMOs. This study estimates European consumers’ willingness-to-pay (WTP) for rice labeled as GM, cisgenic, with environmental benefits (which cisgenesis could provide), or any combination of these three attributes. Data were collected from 3,002 participants through an online survey administered in Belgium, France, the Netherlands, Spain and the United Kingdom in 2013. Censored regression models were used to model consumers’ WTP in each country. Model estimates highlight significant differences in WTP across countries. In all five countries, consumers are willing-to-pay a premium to avoid purchasing rice labeled as GM. In all countries except Spain, consumers have a significantly higher WTP to avoid consuming rice labeled as GM compared to rice labeled as cisgenic, suggesting that inserting genes from the plant’s own gene pool is more acceptable to consumers. Additionally, French consumers are willing-to-pay a premium for rice labeled as having environmental benefits compared to conventional rice. These findings suggest that not all GMOs are the same in consumers’ eyes and thus, from a consumer preference perspective, the differences between transgenic and cisgenic products are recommended to be reflected in GMO labeling and trade policies.

## Introduction

According to the Food and Agriculture Organization (FAO) of the United Nations [1], food supply must drastically increase by 2050 to support the projected global population growth. In fact, the FAO estimates that compared to the world food production in 2008, the world’s food supply must increase by 70% by 2050 [1]. Rice (*Oryza sativa L*.) is the staple food for nearly half of the world’s current population of seven billion people and the staple food of nearly 560 million impoverished consumers in Asia alone [2]. Among the different pathways to increase food production, genetically modified (GM) crops are often presented as a potentially viable, albeit controversial, option. The International Service for the Acquisition of Agri-biotech Applications (ISAAA) predicts that by the end of 2015, GM crops will be sown on approximately 200 million hectares worldwide, distributed over more than 40 countries. The same study estimates that more than 20 million farmers will be using biotechnology by the end of 2015 [3]. Unlike GM corn, soybeans, and cotton, GM rice is not currently commercially produced anywhere in the world. This is partially attributable to the high reluctance towards GM crops in several countries—specifically towards GM rice and wheat. The European Union's (EU) stringent rules on GM crops and labeling, alongside European consumers’ strong aversions to GM food products send signals that discourage seed companies from developing and investing in GM rice and wheat.

Rice and wheat are unique because they are consumed after minimal processing, unlike soybeans and corn which are primarily inputs to animal feeds and biofuels, and cotton which is mainly produced for fibers and for which seeds are processed before human consumption. Thus, advances in breeding for yield and nutritional enhancements along with improvements in biotic and abiotic stress for rice have lagged behind corn, soybeans and cotton for which GM crops have been commercially cultivated since at least 1997. As an example, the rate of annual change of rice yield between 1991 and 2010 has been estimated to be 1% while the rate of annual change for maize yield was equal to 1.5% [4].

Rice production is continually challenged by various evolving diseases and fungi that can reduce output. One of the most destructive rice diseases is rice blast: a fungal disease caused by *Magnaportha grisea*. In the United Sates (US), this fungal disease was identified for the first time in California in 1996 and has gradually spread to the US Mid-South rice-growing region [5]. Its effect can be dramatic if not addressed: throughout the world, annual yield losses caused by rice blast are estimated to be sufficient to feed more than 60 million people [6].

Attempts are underway to foster increased food production with the development of alternative breeding techniques such as cisgenesis. The term “cisgenic” was introduced internationally in 2006 by Schouten, Krens and Jacobsen and is defined as “a crop plant that has been genetically modified with one or more genes (containing introns and flanking regions such as native promoter and terminator regions in a sense orientation) isolated from a crossable donor plant” [7]. Over the last 10 years, however, the term “cisgenic” has been defined differently by several authors [8]. Unlike transgenesis, which is defined as “the genetic modification of a recipient plant with one or more genes from any non‐plant organism or from a donor plant that is sexually incompatible with the recipient plant” [7], the results of cisgenesis could occur naturally over time. The cisgenes are “natural genes” that have not been modified, that have “already been present in the species or in crossable relatives for centuries” and that “could also be transferred by traditional breeding techniques” [7]. Another characteristic of a cisgenic product is that it is exempt of alien DNA such as marker genes or any other vector-backbone genes [9].

Cisgenically engineered plant crops have been developed for several species including apples, barley, and potatoes [9]. In the US, rice breeders along with plant pathologists have begun using cisgenesis to breed a rice blast resistant variety that provides environmental benefits because it requires less pesticides [10]. However, before cisgenic rice can be widely adopted amongst rice producers in the US, they must ensure that export markets, such as the EU, will accept rice bred in this manner. In fact, to date, in the EU and in many countries across the world, cisgenic products fall under very strict GMO legislation. The legal status of cisgenic products is currently debated among scientists and at the EU level [7]. The European Food Safety Authority (EFSA) has issued an opinion on the risks of new breeding techniques such as cisgenesis. EFSA (2012) compared the biosafety of cisgenic plants with that of transgenic and conventionally bred plants and reported that “similar hazards can be associated with cisgenic and conventionally bred plants, while novel hazards can be associated with intragenic and transgenic plants” [12]. The European Commission has established a “New Breeding Techniques” Working Group and is currently, together with the EU Member States, clarifying the legal status of these new breeding techniques [13]. Thus, the future of cisgenic crops is likely to depend on their legal status and on consumer acceptance. The resulting question that remains is both simple and fundamental: would Europeans consumers, most of whom do not accept transgenic crops, be willing to buy and consume cisgenic rice?

### Consumers’ acceptance of cisgenic and transgenic food products in Europe

There is a considerable literature about consumers’ acceptance of GM foods in general [14] or on transgenic food [18], but comparatively little on consumers’ preferences for cisgenic commodities specifically. In addition, to our knowledge, there has not been any study conducted to analyze consumers’ willingness-to-pay (WTP) for cisgenic commodities. Previous studies have shown that European consumers are highly averse to GM food consumption. Among these studies, the European Commission’s Eurobarometer reports show the evolution of European consumers’ attitudes towards biotechnology (including GM food) in the EU. The results of these surveys (conducted in 1991, 1993, 1996, 1999, 2002, 2005 and 2010) [15] suggest that consumers’ opinion on GM food in the EU has evolved in such a manner that they became more averse to GM products over time. In fact, in 1991, 74% of the EU citizens agreed (tended to agree or totally agreed) that genetic engineering research on plants is worthwhile and should be encouraged [15]. In the 2005 Eurobarometer survey, only 27% of the participants supported GM food. In addition, EU citizens viewed GM food as risky, not useful and not morally acceptable and a majority disagreed with the idea that the development of GM food should be encouraged [16]. The latest Eurobarometer on biotechnology (2010) confirmed this negative attitude.

On the other hand, Knight, Mather, Holdsworth and Ermen [14] reported that European consumers were actually willing to consume GM food if the product was both cheaper and provided an environmental benefit (e.g., spray-free fruits). In addition, a study conducted in Switzerland showed that consumers “treat GM foods just like any other type of novel food” [19]. Consistent through all studies is that European consumers’ attitude towards GM food differs across EU Member States. For example, in the 2010 Eurobarometer, the percentage of participants who agreed that the development of GM food should be encouraged ranges from 10% in Greece, Bulgaria and Lithuania to 35% and 36% in the UK and Czech Republic, respectively [17].

Myskja [20] emphasized that one of the main concerns related to transgenic food products is linked to the concept of naturalness and the fact that transgenic food products are seen as “unnatural” by European consumers. Thus, it has been widely assumed in the literature that European consumers may accept cisgenic food products at a higher rate than transgenic food products [20]. Although very few studies [20] have been conducted thus far to test this assumption, it has been revealed that consumers consider cisgenic products as more natural and therefore more acceptable than transgenic products [22, 23]. However, this holds true only when consumers assess the naturalness of a product based on the potential crossing of species borders. European consumers view a cisgenic product as more natural because the breeding process involves genes from the same species compared to a transgenic product in which species borders are crossed. However, both cisgenic and transgenic products remain perceived as unnatural by a majority of EU citizens [24]. The 2010 Eurobarometer survey analyzed the perceptions of the EU citizens on the naturalness of cisgenic and transgenic crops. The results show that a smaller proportion (but still a majority) of them either agreed or tended to agree that cisgenic crops are fundamentally unnatural (52%) compared to 72% for transgenic crops [25]. Some consumers considered cisgenic and transgenic products as equally unnatural because they both require human intervention to be developed. A study conducted in Japan and Austria [24], however, showed that consumers viewed cross-species gene transfers more negatively.

To date, the literature about consumers’ attitudes towards cisgenesis is still sparse and at present little is known about consumers’ WTP for cisgenic food products and if consumers really differentiate cisgenesis from transgenesis. This study analyzes European consumers' attitudes towards cisgenic rice to assess their WTP for rice when labeled specifically as solely GM, or solely cisgenic, or solely as having environmental benefits that cisgenesis could provide, or as various combinations of these three attributes. The results of this study can have significant implications for policy as well as for the production and marketing of cisgenic vis-à-vis transgenic food products. Moreover, the estimation of the WTP values among the labels will be useful in informing policy-makers of the potential impacts of various provisions that could be implemented in the EU. For example, the provisions could require food manufacturers to label their cisgenic food product either as a GM product (i.e., the status quo), or solely as a cisgenic product. Alternatively, the policy could exclude cisgenic products from the current GMO regulation and therefore not require any specific biotechnology labeling but still allow labeling indicating the environmental benefits that could be derived from them.

## Materials and Methods

### Survey design and data collection

A survey was designed to elicit information on EU consumers’ WTP for cisgenic rice, consumers’ attitudes towards cisgenic, transgenic and GM rice and their willingness to consume GM or cisgenic food products, consumption habits, and on demographics (S1 Appendix. Survey questionnaire and treatment presentation.). Ethics approvals regarding the study protocol, participant information materials and research instruments were sought and obtained from the Committee for Medical Ethics UZGent-UGent (Belgium; approval registration number B670201318983, November 2013) and from the Office of Research Compliance Institutional Review Board from the University of Arkansas, Fayetteville (United States of America, approval protocol number 13-10-185, October 2013). The market research company Survey Sampling International (SSI) performed the field work data collection in five European countries during December 2013: Belgium, The Netherlands, Spain, the United Kingdom (UK) and France. In total, 3002 participants completed the survey. Participants were provided information about confidentiality, risks and voluntary participation in the survey, along with instructions that they could quit the survey at any time. Implied consent was obtained if they clicked the arrow to “begin the survey”. The University of Arkansas IRB approved of this implied consent. Only complete surveys were used, so participants who withdrew were not included. The sample was balanced on age and gender. In addition, the sample includes participants from diverse education levels, income levels, and socio-demographic groups. Table 1 shows the socioeconomic characteristics of the sub-sample by country and for the total sample.

**Table 1:** Socioeconomic characteristics by country and for the total sample (%).

	Belgium	France	The Netherlands	Spain	United Kingdom	Total sample
	(N = 500)	(N = 750)	(N = 602)	(N = 399)	(N = 751)	(N = 3002)
*Gender*						
Male	50.2	41.1	44.7	46.4	47.9	45.7
Female	49.8	58.9	55.3	53.6	52.1	54.3
*Education*						
High school or less	49.0	47.2	33.9	36.1	35.8	40.5
Undergraduate^a^	23.8	21.3	45.3	18.3	28.9	28.1
Master degree or PhD	27.2	31.5	20.8	45.6	35.3	31.5
*Living environment*						
Very rural	10.8	12.4	9.1	3.0	7.1	8.9
Somewhat rural	36.2	29.1	23.4	16.3	24.4	26.3
Suburban	23.0	14.5	21.5	8.8	35.1	21.8
Somewhat urban	17.2	25.3	24.9	23.1	18.0	21.8
Very urban	12.8	18.7	20.9	48.9	15.4	21.4
*Net household income* ^b^ ^c^						
Less than €18 000	23.2	25.6	28.4	30.6	25.8	26.5
€18 000 to €34 999	44.2	42.7	37.0	42.4	35.3	39.9
€35 000 to €49 999	20.8	19.1	20.1	16.0	19.0	19.2
€50 000 and more	11.8	12.7	14.5	11.0	19.8	14.5
*Age*						
Less than 30 years old	22.4	19.9	30.6	31.8	21.3	24.4
Between 30 and 39 years old	10.6	19.7	11.0	28.3	19.0	17.4
Between 40 and 49 years old	18.6	26.4	16.9	25.6	22.0	22.0
Between 50 and 59 years old	21.6	20.3	20.1	11.0	20.2	19.2
60 years old or more	26.8	13.7	21.4	3.3	17.4	17.0
*Household composition*						
Respondent lives alone	20.8	21.6	24.9	6.5	26.6	20.1
One or more children aged less than 7	8.6	16.0	11.6	18.3	16.0	14.2

All results are presented in percent save for the mean age that is presented in years.

^a^ This education category encompasses participants who have obtained a degree equivalent to 3 or 4 years of additional studies after the completion of high school.

^b^ In the United Kingdom, all monetary units were presented in equivalent pounds £.

^c^ Net income represents the income after taxes.

The survey was designed to allow collection of consumer responses that would reveal their WTP for a 2.25 kg bag of rice under different information scenarios. This WTP elicitation was based upon the multiple price list (MPL) format [26]. The MPL format can be used to estimate the WTP for commodities or products, as well as to elicit risk attitudes and individual discount rates. One of the main benefits of the MPL is that it is straightforward to implement and easy for participants to understand. However, the MPL estimates intervals instead of ‘point’ valuations. In this study, the MPL questions were presented one by one and there was no possibility for the participant to go back and change her/his answer. This approach was used to mitigate possible bias from framing effects and switching behavior, which have previously been recognized as possible drawbacks from MPL [27].

Participants were asked to choose between a 2.25 kg bag of a conventionally bred rice variety and an alternative rice variety (described under different information scenarios), presented as Variety A. The price of the alternative rice variety (used as baseline) was constant (€2.25) for a 2.25 kg bag of rice, while the price of the conventional variety decreased from €50 to €0.5 with intermediate values at €20, €15, €10, €8, €5, €3, €2.25 and €2.

In an attempt to mitigate the risk of the so-called hypothetical bias, which can be common in hypothetical surveys or polls, a cheap talk script was included in the introduction of the survey (S1 Appendix. Survey questionnaire and treatment presentation.). Studies have shown that participants in a hypothetical survey tend to state higher WTP than when faced with actual decisions [28]. In order to reduce that hypothetical bias, “cheap talk” can be incorporated at the beginning of a survey [29] to inform participants about this potential bias, and ask them expressly to avoid it.

All participants responded to three rounds of questions. In the first round, the alternative rice variety (Variety A) was presented as either GM, or cisgenic or as a variety providing environmental benefits. Thus participants received one type of information (cisgenic, GM or environmental benefits) in the first round. Table 2 shows the description that was presented for each of the attributes of Variety A.

**Table 2:** Descriptions of Variety A attributes.

GM	No additional information was given with respect to the GM attribute except it was a GM product.
Environmental Benefits	“New breeding techniques can result in a rice variety that is resistant to rice blast disease and that would not require fungicide sprays. Rice blast is a disease that decreases yields and increases Greenhouse Gas emissions because of the fungicide sprays that are required to treat the disease. The variety A would not require fungicide applications.”
Cisgenic	“Cisgenic rice is bred using a process in which genes are transferred between crossable organisms (the same species or closely related species). The same result could be obtained by cross-breeding that occurs in nature or by traditional breeding methods but it would require a longer time frame.”

In each successive round, participants were presented additional information about the alternative rice variety. In the second round, two out of the three attributes of the alternative rice (including those received in round 1) were presented to the participants, increasing the participants’ knowledge from the first round. In the third round all participants received the full information on all three characteristics: GM, cisgenic, and the associated environmental benefits. These three attributes describing Variety A were provided to participants in different orders, resulting in 15 possible orders—further referred to as “treatments”—depending on the sequence in which the three types of information were given. Participants were randomly assigned to one of the treatments. For each country, an equal number of participants were assigned to each treatment. Table 3 illustrates these 15 treatments, depending on the sequence in which the three types of information were given.

**Table 3:** Ordering of information treatments for Variety A.

Treatment	Round	Information
1	1	GM
2	1	Cisgenic
3	1	Environmental Benefits
4	2	Environmental Benefits—Cisgenic
5	2	Environmental Benefits—GM
6	2	Cisgenic—GM
7	2	Cisgenic—Environmental Benefits
8	2	GM—Environmental Benefits
9	2	GM—Cisgenic
10	3	GM—Cisgenic—Environmental Benefits
11	3	GM—Environmental Benefits—Cisgenic
12	3	Cisgenic—GM—Environmental Benefits
13	3	Cisgenic—Environmental Benefits—GM
14	3	Environmental Benefits—Cisgenic—GM
15	3	Environmental Benefits—GM—Cisgenic

### Interval regression modeling

An interval regression model was specified to estimate the premium consumers would be willing to pay for conventional rice to avoid the alternative rice. However, these premiums were not directly observed. For each participant, the WTP for the conventional rice instead of the alternative rice was observed as an interval [Y_i1_, Y_i2_] for participant *i* where Y_i1_ < Y_i2_. Assuming participants are rational, the actual WTP, Y_i_*, lies in this interval (including the lower boundary). To control for the impact of the hypothesized conditioning variables (regressors), we specified a linear WTP model as:

$$Y_{i}^{*}=\beta_{0}+\beta_{1\;}Treatment+\beta_{2\;}Agecategory+\beta_{3\;}Childlt7+\beta_{4\;}Education+\beta_{5\;}Income+\varepsilon_{i}$$ (1)

where ε_i_ is normally distributed with mean zero and variance σ^2^. The five listed regressors (Treatment, Agecategory, Childlt7, Education and Income) are all categorical variables (Table 4). The impacts of these variables are identified by assuming one of the categories for each regressor takes on the value zero and so becomes the base level for that variable. Consequently the parameter β_0_ (the intercept) is the baseline WTP of a participant less than 30 years old, with no children less than 7 years old in their household, with an education equivalent to high school or less and with an annual net household income less than €18,000. The **β**j are vectors that represent the deviations from the base level of each of the five categorical variables.

**Table 4:** Interval regression results of WTP to consume conventional rice instead of the alternative, by country^1^.

	Belgium	France	The Netherlands	Spain	United Kingdom
***Intercept***	10.68**	29.83**	16.02**	16.97**	18.46**
***Treatment***					
GM					
Cisgenic	-7.63**	-15.87**	-6.82**	-1.97	-7.68**
Env. Benefits	-9.82**	-44.88**	-9.15**	-15.43**	-13.93**
Cisgenic—GM	-4.61	-12.60**	-2.35	1.92	-6.03*
Cisgenic Env. Benefits	-8.89**	-23.05**	-9.48**	-10.25**	-10.55**
GM—Cisgenic	0.92	-2.54	-2.17	-1.23	0.64
GM—Env. Benefits	-2.16	-9.27**	-1.13	-3.96	-5.53**
Env. Benefits—GM	-5.79	-26.69**	-7.08**	-11.50**	-5.42
Env. Benefits—Cisgenic	-9.72**	-42.88**	-9.17**	-14.00**	-13.09**
Cisgenic—GM—Env. Benefits	-7.27**	-17.62**	-4.30	-5.96	-8.86**
Cisgenic- Env. Benefits—GM	-6.81*	-19.05**	-9.56**	-7.21*	-7.59**
GM—Cisgenic—Env. Benefits	-4.28*	-9.79**	-3.82	-9.16**	-4.10*
GM—Env. Benefits—Cisgenic	-2.25	-12.60**	-0.51	-4.42	-5.02**
Env. Benefits—GM—Cisgenic	-5.78	-24.45**	-3.97	-11.32**	-5.56*
Env. Benefits—Cisgenic—GM	-10.13**	-32.24**	-7.67**	-11.05**	-9.59**
***Age categories***					
Less than 30 years old					
Between 30 and 39 years old	3.11	0.92	1.76	-2.24	-0.11
Between 40 and 49 years old	-1.68	3.15	-5.04*	-5.21*	-4.06
Between 50 and 59 years old	3.18	6.33	-1.16	-1.24	-2.61
60 years old or more	3.03	1.91	-0.99	8.64	-4.72
***Children less than 7 in the household***					
Yes	0.50	6.28*	1.01	-2.78	-0.34
***Education***					
High school or less					
Undergraduate	1.45	2.62	3.60*	1.16	2.57
Master degree or PhD	3.43	5.84*	0.56	-0.41	5.90**
*Income*					
Less than €18 000					
€18 000 to €34 999	0.99	2.06	-4.20*	0.08	-0.89
€35 000 to €49 999	-1.11	5.23	-5.41**	0.82	-4.95*
€50 000 and more	-2.47	3.86	1.32	-0.26	0.80
*Sigma* ^2^	20.55	31.25	19.82	21.94	23.35

^1^ In the United Kingdom, all monetary units were presented in equivalent pounds £.

^2^ Sigma is an estimate of the error term, ε_i_, in equation (1).

* Indicates statistical significance at the 5% level.

** Indicates statistical significance at the 1% level.

While previous WTP studies have shown that gender significantly affects the WTP and acceptance for biotechnology [30], gender was insignificant (p>0.05) in all our models estimated for each of the five countries. Similarly, the variables “living environment” and “size of the household” were insignificant in preliminary estimation. Due to the lack of significance, these three variables were not included in the estimated models. The variables “*Agecategory*”, “*Education*”, “*Childlt7*” and “*Income*” were kept in the model because these were significant (p<0.05) in at least one of the five studied countries.

The variable “Treatment” (Eq 1) indicates the information that the participants received (regarding rice Variety A) as well as the order in which information was provided to them. By taking into account the type of information received and the order in which information was provided, participants were assigned to three of the 15 different “treatments” (one treatment for each round) (Table 3). The WTP questions were asked in three information rounds for each participant.

In the estimation process it can be argued that consumers who would not consume a GM product under any circumstances should have been eliminated from the sample. However, participants were presented information about cisgenic breeding sequentially in the survey instrument. Thus, it was felt that the information sets had likely influenced attitudes toward consumption, and that aversion to GM would be captured in the WTP amounts. Therefore, all participants were included in the sample and as a result, the sample is a panel with three WTP values to avoid or pay for the alternative rice obtained from each participant, corresponding to the three information rounds.

The coefficients in Eq 1 are estimated by maximum likelihood methods. The likelihood function from Eq 1 is the product of the probabilities of the observations lying in the observed interval (so for individual *i*, Pr (Yi1 ≤ Yi* < Yi2)). All interval values were adjusted by subtracting €2.25, the price at which the conventional rice is presented to the participants. An observation is right censored if the participant is willing to pay a premium of €47.75 (€50-€2.25) or more to avoid the alternative rice, so then the probability is Pr (€47.75 ≤ Yi*). If the observation is left censored the participant is willing to pay €1.75 (€0.5-€2.25 = €-1.75) or more to consume the alternative rice instead of the conventional rice, so then the probability is Pr (Yi* ≤ €-1.75). The maximum likelihood estimates were obtained from using the interval regression command “INTREG” in STATA 13. Finally, because the sample was by nature panel data, clustered robust standard errors were estimated to account for the correlation existing in the error term (ε_i_) among the three observations for a given participant.

## Results and Discussion

On average, 36% of all the participants are willing to consume a GM food product with values ranging from 23% in France to 47% in the UK (Table 5). Across all countries, the willingness to consume a cisgenic product is 38% with values ranging from 27% in France to 52% in Spain. Participants from France are the least willing to consume a GM and a cisgenic food product, with 31% and 26% of the French participants being not willing to consume a GM or cisgenic food product, respectively. Across countries a larger proportion of participants (38% versus 36%) were willing to consume cisgenic than GM but this difference was not significant at 0.05. The Netherlands and UK had contrary outcomes but the differences in the “Yes” proportions within both the Netherlands and UK were not significant at 0.05. The differences for France and Spain were significant at 0.05.

**Table 5:** Willingness to consume GM and cisgenic food by country (%).

	Belgium	France	Netherlands	Spain	United Kingdom	Total sample
	(N = 500)	(N = 750)	(N = 602)	(N = 399)	(N = 751)	(N = 3002)
*Willingness to consume GM food*						
No	10	31	15	11	15	17
Yes	38	23	32	46	47	36
Not enough information	52	46	53	43	39	46
*Willingness to consume cisgenic food*						
No	9	26	10	8	10	14
Yes	38	27	30	52	46	38
Not enough information	53	47	60	40	44	49

The percentage of participants who indicated that they did not have enough information to decide whether they would be willing to consume a cisgenic or a GM food product was relatively high (46% of the participants for GM food and 49% for cisgenic food). Thus there could be wider acceptance for GM and cisgenic products but promotional or at least educational campaigns are greatly needed to inform consumers about each. In addition, in all countries but Spain, the percentage of “Not enough information” for the question regarding the willingness to consume a cisgenic or a GM food product is higher for the cisgenic product. Furthermore, the percentage of “No” is always smaller for the cisgenic product than for the GM product. This shows that participants are less familiar with or less knowledgeable about cisgenic food products than GM food products, but also somewhat less aversive toward cisgenic. This finding is consistent with previous results [24] showing that consumers have a more negative attitude when they are more familiar with the method of genetic modification.

The coefficient estimates of the interval regression (Eq 1) are presented in Table 4 Due to important differences observed across countries, separate regression models were estimated for each country, all having the same independent variables as in Eq 1.

The mean WTP to avoid a 2.25 kg bag of GM rice for the baseline group was estimated to be €10.68 in Belgium, €16.02 in the Netherlands, €16.97 in Spain, €18.46 in the UK and €29.83 in France (Table 4). Thus, these values represent the amounts of money participants are willing to pay extra to obtain conventional rice instead of GM rice. These results illustrate the variation amongst consumers in the EU to avoid GM food products. French consumers have the largest aversion and Belgian consumers have the smallest aversion to GM rice. The findings for France are consistent with current attitudes towards GM products in Europe since France is the only one of the five countries in this study to use the safeguard clause to avoid the cultivation of GM food products (more specifically to avoid the cultivation of maize MON810) [31]. On the other hand, it was expected that Spanish consumers would have a lower aversion to GM than in the other countries since Spain is the only one out of the five studied countries to currently grow a GM crop (MON810 maize). For all countries except Spain, WTP to avoid cisgenic rice is lower than for GM rice illustrating again that participants are less averse for rice labeled as cisgenic than for rice labeled as GM.

These results indicate that participants are willing to pay roughly four to thirteen times more than the price of GM rice (€2.25) to avoid GM across all countries. Two possible reasons could explain these seemingly high figures. First, in the online questionnaire, participants were not allowed to decline a rice purchase: they were forced to pick one of the two prices provided to them. Thus, participants who are not willing to consume a GM or a cisgenic food product had no other choice than to pay €50 to avoid it. As mentioned above, there was consideration of whether to restrict the analysis of WTP to participants who did not reject consumption of GM or cisgenic rice. Because participants were presented information about cisgenic breeding sequentially in the survey instrument, it was felt that the information sets had likely influenced attitudes toward consumption, and that aversion to GM would be captured in the WTP amounts. Therefore all participants were included in the sample to estimate the WTP model. However, after the MPL questions, participants were asked if they would be willing to consume a GM food product if it were available: the proportion of consumers *not* willing to consume a GM food product was approximately 10% of the participants in Belgium, 31% in France, 15% in the Netherlands, 11% in Spain and 15% in the UK. Second, previous non-hypothetical studies have shown that European consumers are willing to pay high premiums to avoid consuming GM food. As an example, Lusk [32] found that French consumers are willing to pay a premium of up to $9.18 per pound of beef to avoid consuming beef fed with GM corn in a non-hypothetical setting.

The French participants are willing to pay the least to avoid the alternative when the alternative is presented as “with environmental benefits” compared to the alternative rice presented as “GM” or as “cisgenic” (Table 4). In fact, in France the coefficient for “environmental benefits” is -44.88, meaning that French participants were willing to pay €15.05 more (29.83+(-44.88)) to obtain the alternative rice described as having environmental benefits compared to the conventional rice. The findings with respect to consumer interest in the environmental benefits are generally consistent with the results of the 2014 Eurobarometer report on EU “citizen attitudes towards the environment” [33]. This report indicates that a much higher share of French citizens (35%) rated “agricultural pollution (e.g. from the use of pesticides)” among their top-five environmental issues worried about. By contrast, this share was only 28, 19, 23 and 33% among citizens in Belgium, United Kingdom, The Netherlands and Spain, respectively. Furthermore, the 2014 Eurobarometer found that there was little difference across this study’s citizens when asked “Are you willing to buy environmentally friendly products even if they cost a little bit more” with 78, 80, 82, 77 and 73% of citizens in France, Belgium, UK, The Netherlands and Spain indicating yes, respectively. However, coupled with another question from the Eurobarometer [17] “Do you agree that GM food does no harm to the environment” there are large differences with the French sediment agreeing with that statement the least at 15% with 30, 25, 22, and 28% of citizens in Belgium, UK, The Netherlands and Spain indicating yes, respectively. Given these two pieces of information it would appear that the French citizens in this study may have viewed GM as causing more harm to the environment than other countries citizens and as such, while there were not large differences in WTP for environmentally friendly products, there were large differences in WTP for GM possibly as a result of differences in perception on how GM effects the environment.

In Belgium and in the Netherlands, the smallest WTP to avoid the alternative rice is found in round 3 (when all information has been provided). In all five countries, consumers are willing to pay the highest premium to avoid consuming the alternative rice when they are only provided with the information that this rice is GM.

In the model for France, 13 of the 14 regression coefficients for the variable *treatment* are statistically significant at the 5% level and all coefficients are negative. Once again, this underscores that French consumers are sensitive to all of the information sets (positive for environmental and negative for cisgenic and GM). In all five countries, the coefficients for the category “Environmental benefits” of the variable “Treatment” are statistically different from 0 at the significant level p<0.05. This shows that, as expected, consumers are willing to pay more for a product labeled as having environmental benefits compared to a product labeled as GM only.

### Labeling Implications

**Fig 1:**
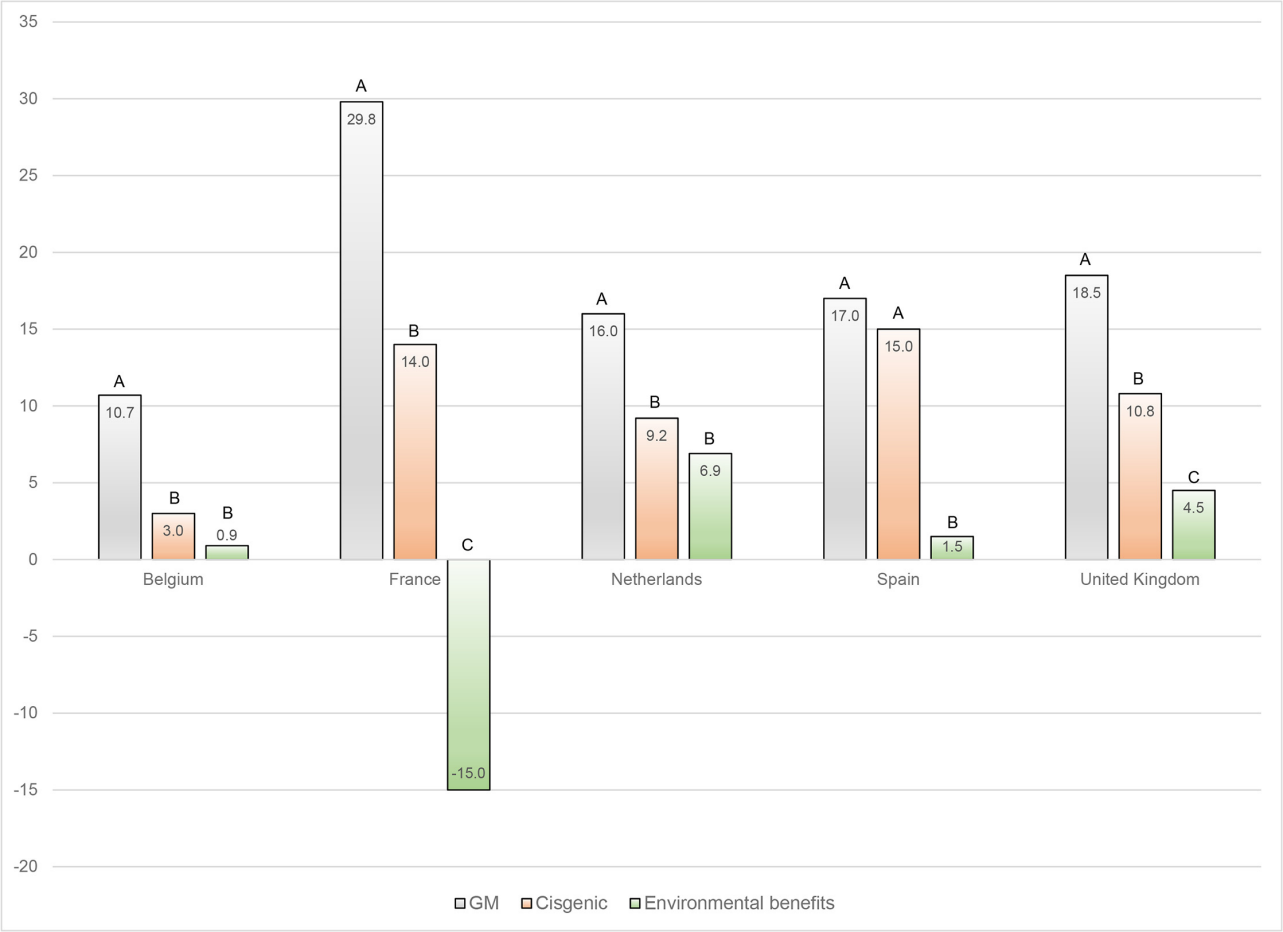
Willingness-to-pay [€] by country for conventional rice instead of rice labeled as GM, cisgenic or with environmental benefits. The bars represent the premium or discount that consumers are willing to pay for conventional rice in order to avoid rice labeled as GM (grey bars), as cisgenic (red bars) or as having environmental benefits (green bars). All results are in Euros. The UK participants answered in GBP but the values above are converted to Euros using an exchange rate of 0.8431 GBP per Euro. For a given country, bars identified by the same letter are not statistically different from one another at the (p<0.05) significance level.

Fig 1 depicts the magnitudes of the first-round treatment effects in which the variable “*treatment*” corresponds to the information provided in round 1 only (GM, cisgenic, or environmental benefits). These three treatments provide important information for policy development and decision-making. These first-round treatment effects directly estimate the differences in European consumers’ WTP for a 2.25 kg bag of rice as a function of GMO characteristics and environmental benefits claimed. The differences in WTP among the labels are useful in informing policy-makers of the potential impacts of various provisions that could be implemented in the EU. As previously mentioned, such provisions could require food manufacturers to label their cisgenic food product as a GM product (scenario 1), or solely as cisgenic (scenario 2), or the policy could exclude cisgenic products from the current GMO regulation (and therefore not require any specific biotechnology labeling) but still allow labeling indicating environmental benefits (scenario 3).

Scenario 1 illustrates the current EU policy towards cisgenic products: cisgenic products fall under the EU GMO regulation and have to be labeled as such if the product consists of or contains more than 0.9% of GM product. In accordance with EU Regulation 1830/2003, the phrase “This product contains genetically modified organisms” or “This product contains genetically modified [name of organism(s)]” is mandatory information on the product label [34].

The second scenario assumes that the current EU policy is modified to account for the specific type of GM food product; that is, differentiating cisgenics from transgenics. In this hypothetical situation, food companies might still be required to label their product as cisgenic but not as GM. Companies would be allowed to state that the product is bred cisgenically and the words “genetically modified” would not have to appear on the label.

Finally, in the third scenario, it is assumed that cisgenic breeding is added to the Annex 1B in EU Directive 2001/18 [35] concerning the ‘deliberate release into the environment of genetically modified organisms’. The annex 1B is a list of genetic modification processes that do not fall under the EU GMO policy. In this case, there would be no specific labeling regulation concerning the breeding process. Therefore, if the commodity resulting from cisgenesis provides environmental benefits such as a reduction in the use of pesticides, it is assumed that food manufacturers would be able to label their cisgenic food product as “product with environmental benefits”.

Fig 1 highlights several interesting findings. First, in every country except Spain, consumers are willing to pay significantly (p < 0.05) more to avoid GM than cisgenic rice. In every case, consumers are willing to pay less to avoid the product labeled as cisgenic, compared to the base (conventionally bred rice), *ceteris paribus*. This emphasizes an important finding: consumers in France, Belgium, The Netherlands and the UK view cisgenic products as a significantly different type of GM product relative to a transgenic product. On average, across all countries, consumers are willing to pay €7.99 more (scenario 1—scenario 2) to avoid consuming a product labeled as GM compared to a product labeled as cisgenic. This implies that the mandatory labeling of cisgenic products as GM would negatively affect purchasing decisions for cisgenic products. In Spain, there are no statistically significant differences in the WTP estimates for rice labeled as cisgenic and as GM. This result is not surprising given that Spain currently produces GM crops. This also confirms the results from the Eurobarometer studies showing that Spanish consumers perceive the least problem with GM crops. Spanish consumers might either avoid GM products at all or they might view GM products as less of an issue and thus cisgenic products are lumped into a category that they already accept to some degree. So this may be the reason why Spanish consumers do not differentiate between the two. In the third scenario (rice labeled as “with environmental benefits”), consumers in each country have the lowest WTP to avoid the product compared to the base of conventionally bred rice. In all five countries, the WTP to avoid consuming rice labeled “with environmental benefits” is statistically different (p<0.05) than the WTP to avoid consuming rice labeled as GM. Furthermore, in Spain, France and the UK, the WTP to avoid consuming rice labeled as having environmental benefits is also statistically different (p<0.05) from the WTP to avoid rice labeled as cisgenic. Alternatively, consumers in the Netherlands or in Belgium do not differentiate between rice labeled as cisgenic or as having environmental benefits but they do differentiate between rice labeled as GM as opposed to cisgenic or with environmental benefits.

Interestingly, while French consumers have the highest aversion to GM and therefore are willing to pay the highest premium to avoid rice labeled as GM, they also have the highest WTP for rice labeled “with environmental benefits”. This results in a negative WTP € (-15.05) to avoid rice labeled as having environmental benefits, which means that French consumers are actually willing to pay a premium to obtain rice labeled as having environmental benefits compared to conventional rice. In other words, French consumers are willing to pay €15.05 more to have rice labeled as having environmental benefits compared to conventional rice.

These findings highlight the important economic implications of different labeling requirements in Europe. Moreover, as it has been emphasized on several occasions, the consequences of labeling requirements are not only economic [37]. In fact, it is argued that the exclusion of cisgenesis from the GMO regulation will strengthen European consumers’ aversion to transgenic food products and further delay the acceptance for such products [36]. In the US, the debate is currently going on regarding mandatory labeling for GM foods, opponents arguing that this will lead to a stigmatization of GM foods and pointed out as a step backward [37].

## Conclusion

In contrast to previous experimental and econometric analyses of consumers’ WTP for GMO’s in Europe [11,20], this study examined if there are differences in acceptance and WTP for cisgenic and GM food and addressed labeling issues associated with possible differentiation between cisgenic and transgenic products and the resulting environmental benefits. This study provides useful information for policy-makers, researchers and producers. First, the results inform policy-makers on consumers’ opinions and help policy-makers design and implement appropriate legislation for the new varieties resulting from new breeding techniques, and more particularly from cisgenesis. This study indicates that consumers generally differentiate cisgenic and transgenic products in both perception and WTP. This suggests that specific policies tailored to cisgenesis could be developed, and that cisgenesis and transgenesis could be treated differently from a policy standpoint. There also appears to be a need for policy-makers as well as for industries to inform consumers about the difference between cisgenics and transgenics given that about half of the participants claimed they did not have enough information to make an informed decision regarding their willingness to consume a GM or a cisgenic food product.

Second, these results are relevant for industries by informing them of the potential market for cisgenic foods. This also sends a less nebulous signal to researchers (both public and private) and seed companies as it appears that cisgenically bred products are acceptable to some EU consumers. In general consumer interest in the environmental impact of food choice is increasing [33]; this paves the way for a stronger acceptance of foods that provide environmental benefits in the future. However, for private companies to develop cisgenic products, the potential cost reductions in producing these products will have to be large enough to compensate for the lower price that consumers are willing to pay for them compared to conventionally bred products. This study indicated that the price that consumers are willing to pay for a 2.25kg bag of cisgenic rice might on average be between €3.1 (in Belgium) and €15.0 (in Spain) lower than the price of conventional rice.

Nevertheless, one of the most important findings in this study is the evidence that European consumers actually differentiate cisgenic from transgenic products. In addition, this study confirms the hypothesis widely assumed in the literature that European consumers may accept cisgenic food products more readily than transgenic food products [21]. These findings suggest that not all GMOs are the same in consumers’ eyes and thus, from a consumer preference perspective, GMO labeling and trade policies could consider the differences between transgenic and cisgenic products. Our hope is that this study will help open the door for many more studies and analyses of consumers’ attitudes and WTP for cisgenic products.

## Supporting Information

S1 Appendix Survey questionnaire and treatment presentation. (PDF)

S1 Dataset (CSV)
