# Supplementary material for: Revisiting GMOs: Are There Differences in European Consumers’ Acceptance and Valuation for Cisgenically vs Transgenically Bred Rice?
Source: PLoS One. 2015 May 14;10(5):e0126060. doi: 10.1371/journal.pone.0126060 (PMC4431710; doi:10.1371/journal.pone.0126060)
Supplement: S1 Appendix — (PDF) [file pone.0126060.s001.pdf]

S1 Appendix. Survey questionnaire and treatment presentation.

The survey questionnaire below is presented with Headings for the Information Blocks, along with question numbers that use the form Block#.Question#, (e.g. Q.1). The headings and question numbers as well as italic text are not part of the survey that is seen by the respondent but are used here for organization.

There are 7 information sets presented below, in which respondents make choices between two rice varieties. Each respondent will see 3 of those information sets. There are 6 Treatments consisting of 3 information sets as listed in the following table. . The treatments are randomly assigned to a respondent in a manner that ensures the same number of presentations of each treatment.

Random Treatment	Information Set 1	Information Set 2	Information Set 3
**1**	Cisgenic	GM Cisgenic	GM Cisgenic Environmental
**2**	Cisgenic	Cisgenic Environmental	GM Cisgenic Environmental
**3**	GM	GM Cisgenic	GM Cisgenic Environmental
**4**	GM	GM Environmental	GM Cisgenic Environmental
**5**	Environmental	GM Environmental	GM Cisgenic Environmental
**6**	Environmental	Cisgenic Environmental	GM Cisgenic Environmental

Introduction

Q1.1: Consumer buying behavior towards rice

Description*:* In the present study we are interested in how you make decisions on purchasing food products. This on-line survey should require 10 minutes to complete. Your opinions are important to us and we hope that you will take the time to give us your insights on your priorities.

Risks and Benefits: Your participation will assist in the advancement of knowledge of consumer choice behavior. There are no anticipated risks to participating in this study.

Voluntary Participation: Your participation in the research is completely voluntary.

Confidentiality*:* Your responses on the survey will be recorded anonymously. No identifying personal information will be collected on the survey. Only basic demographic information (i.e. age, gender, education etc.) will be collected.

Right to Withdraw: You are free to refuse to participate in the research and to stop filling out the survey at any time. If you have questions or concerns about this study, you may contact xxxxxxxxx. For questions or concerns about your rights as a research participant, please contact xxxxxxxxx, the University's Compliance Coordinator, at xxxxxxx or by e-mail at xxxxxxxxx.

Thank you for your participation! Click the arrow below to begin the survey.

Q1.2
Studies show that people tend to act differently when they face hypothetical decisions. In other words, they say one thing and do something different. For example, some people state a price they would pay for an item, but when this item becomes available in a grocery store, they will not pay the price they said they would pay. We want you to behave in the same way that you would if you really had to pay for the product and take it home.

For the following questions, assume that you are buying non fragrant long grain white rice and need a 2.25 kg bag. Three comparison sets of rice varieties with different characteristics will be presented. In each set, two rice varieties will be described and we want you to indicate your preferences between the two varieties at different prices.

Cisgenic Rice Information Set

Q2.1
Cisgenic Rice is bred using a process in which genes are transferred between crossable organisms (same species or closely related species). The same result could be obtained by cross-breeding that occurs in nature or by traditional breeding methods but it would require a longer time frame.

In the next questions we want you to indicate your preferences between two rice varieties at the indicated prices:

(1) a Cisgenic rice variety
(2) a Conventionally-bred rice variety

Q2.2 *(The same choices are repeated with decreasing prices for Conventional variety until Conventional variety is chosen: € 20, € 15, € 10, € 8, € 5, € 3, € 2.25, € 2, € 1, € 0.5)*
Assume that you are buying non fragrant long grain white rice and need a 2.25 kg bag. Indicate

your preferences between two rice varieties at the indicated prices:

(1) a Cisgenic rice variety
(2) a Conventionally-bred rice variety

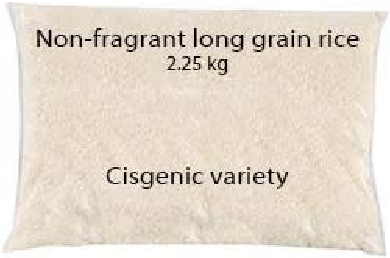

Cisgenic variety
€2.25

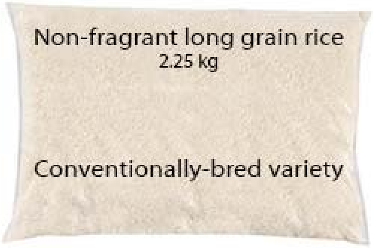

Conventional variety
€50.00

GM Cisgenic Rice Information Set

Q3.1
Cisgenic rice is a Genetically Modified (GM) rice variety that is bred using a process in which genes are transferred between crossable organisms (same species or closely related species). The same result could be obtained by cross-breeding that occurs in nature or by traditional breeding methods but it would require a longer time frame.
In the next questions we want you to indicate your preferences between two rice varieties at the indicated prices:
(1) a Genetically Modified (GM) Cisgenic rice variety
(2) a Conventionally-bred rice variety

Q3.2 *(The same choices are repeated with decreasing prices for Conventional variety until Conventional variety is chosen: € 20, € 15, € 10, € 8, € 5, € 3, € 2.25, € 2, € 1, € 0.5)*
Assume that you are buying non fragrant long grain white rice and need a 2.25 kg bag. Indicate your preferences between two rice varieties at the indicated prices:

(1) a Genetically Modified (GM) Cisgenic rice variety
(2) a Conventionally-bred rice variety

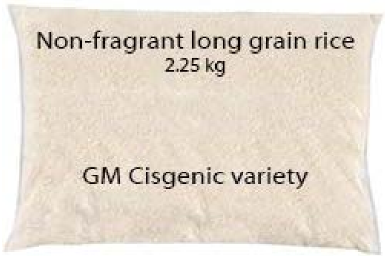

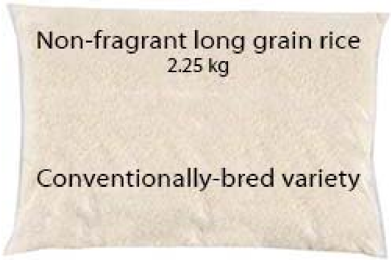

Genetically Modified (GM) Cisgenic variety
€2.25

Conventional variety
€50.00

GM Cisgenic Environmental Rice Information Set

Q4.1
Cisgenic rice is a Genetically Modified (GM) rice variety that is bred using a process in which genes are transferred between crossable organisms (same species or closely related species). The same result could be obtained by cross-breeding that occurs in nature or by traditional breeding methods but it would require a longer time frame.

Cisgenic breeding can result in a rice variety that is resistant to rice blast disease and would not require fungicide sprays. Rice blast is a disease that decreases yields and increases Greenhouse Gas emissions because of the fungicide sprays that are required to treat the disease.

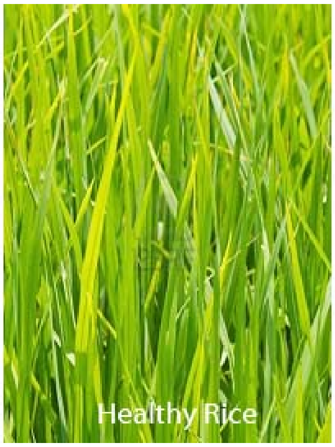

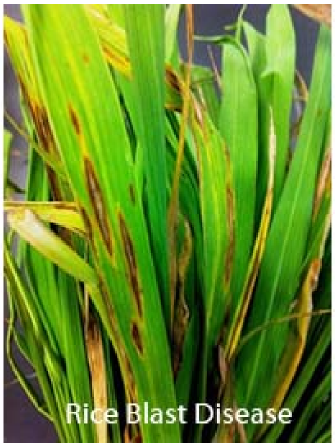

In the next questions we want you to indicate your preferences between two rice varieties at the indicated prices:

(1) a Genetically Modified (GM) Cisgenic rice variety that would not require fungicide

applications
(2) a Conventionally-bred rice variety

Q4.2 *(The same choices are repeated with decreasing prices for Conventional variety until Conventional variety is chosen: € 20, € 15, € 10, € 8, € 5, € 3, € 2.25, € 2, € 1, € 0.5)*
Assume that you are buying non fragrant long grain white rice and need a 2.25 kg bag. Indicate your preferences between two rice varieties at the indicated prices:

(1) a Genetically Modified (GM) Cisgenic rice variety that would not require fungicide applications
(2) a Conventionally-bred rice variety

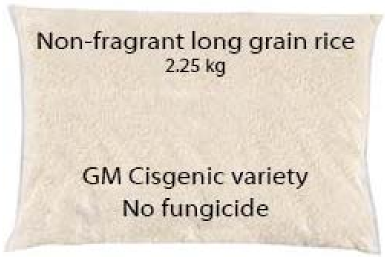

Genetically Modified (GM) Cisgenic variety No Fungicide
€2.25

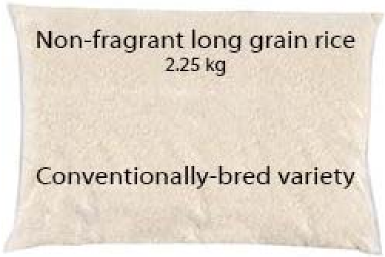

Conventional variety
€50.00

Cisgenic Environmental Rice Information Set

Q5.1
Cisgenic Rice is bred using a process in which genes are transferred between crossable organisms (same species or closely related species). The same result could be obtained by cross-breeding that occurs in nature or by traditional breeding methods but it would require a longer time frame.

Cisgenic breeding can result in a rice variety that is resistant to rice blast disease and would not require fungicide sprays. Rice blast is a disease that decreases yields and increases Greenhouse Gas emissions because of the fungicide sprays that are required to treat the disease.

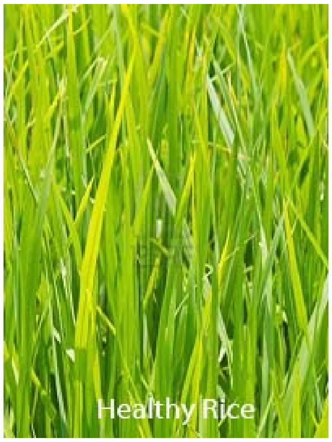

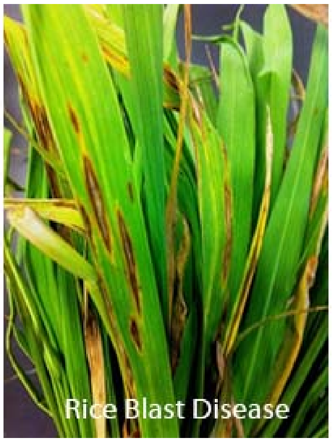

In the next questions we want you to indicate your preferences between two rice varieties at the indicated prices:

(1) a Cisgenic rice variety that would not require fungicide applications
(2) a Conventionally-bred rice variety

Q5.2 *(The same choices are repeated with decreasing prices for Conventional variety until Conventional variety is chosen: € 20, € 15, € 10, € 8, € 5, € 3, € 2.25, € 2, € 1, € 0.5)*
Assume that you are buying non fragrant long grain white rice and need a 2.25 kg bag. Indicate your preferences between two rice varieties at the indicated prices:

(1) a Cisgenic rice variety that would not require fungicide applications
(2) a Conventionally-bred rice variety

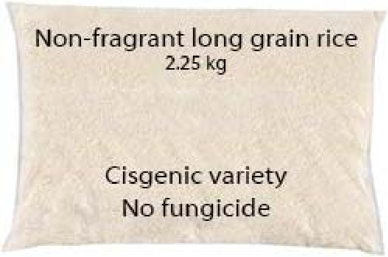

Cisgenic variety
No Fungicide
€2.25

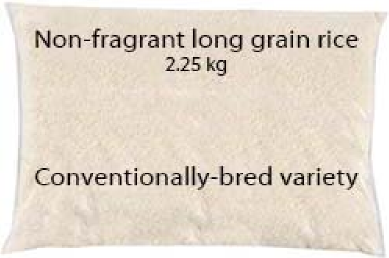

Conventional variety
€50.00

GM Rice Information Set

Q6.1
In the next questions we want you to indicate your preferences between two rice varieties at the

indicated prices:
(1) a Genetically Modified (GM) rice variety
(2) a Conventionally-bred rice variety

Q6.2 *(The same choices are repeated with decreasing prices for Conventional variety until Conventional variety is chosen: € 20, € 15, € 10, € 8, € 5, € 3, € 2.25, € 2, € 1, € 0.5)*
Assume that you are buying non fragrant long grain white rice and need a 2.25 kg bag. Indicate your preferences between two rice varieties at the indicated prices:

(1) a Genetically Modified (GM) rice variety
(a) a Conventionally-bred rice variety

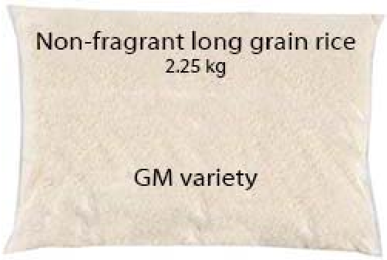

Genetically Modified (GM) variety
€2.25

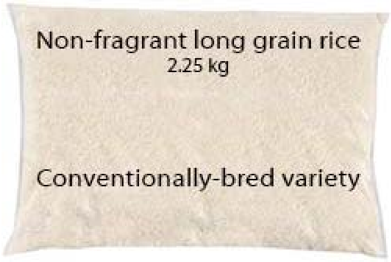

Conventional variety
€50.00

GM Environmental Rice Information Set

Q7.1
Genetic Modification (GM) breeding can result in a rice variety that is resistant to rice blast disease and would not require fungicide sprays. Rice blast is a disease that decreases yields and increases Greenhouse Gas emissions because of the fungicide sprays that are required to treat the disease.

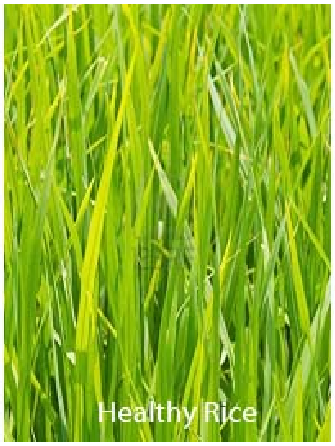

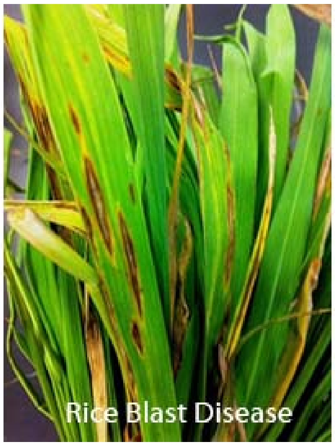

In the next questions we want you to indicate your preferences between two rice varieties at the indicated prices:

(1) a Genetically Modified (GM) rice variety that would not require fungicide applications
(2) a Conventionally-bred rice variety

Q7.2 ***(The same choices are repeated with decreasing prices for Conventional variety until Conventional variety is chosen: € 20, € 15, € 10, € 8, € 5, € 3, € 2.25, € 2, € 1, € 0.5)***

Assume that you are buying non fragrant long grain white rice and need a 2.25 kg bag. Indicate your preferences between two rice varieties at the indicated prices:

(1) a Genetically Modified (GM) rice variety that would not require fungicide applications
(2) a Conventionally-bred rice variety

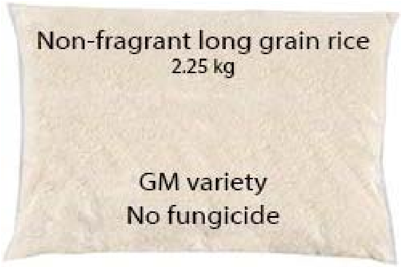

Genetically Modified (GM) variety
No Fungicide
€2.25

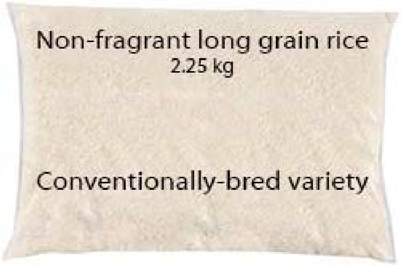

Conventional variety
€50.00

Environmental Rice Information Set

Q8.1
New breeding techniques can result in a rice variety that is resistant to rice blast disease

and would not require fungicide sprays. Rice blast is a disease that decreases yields and increases Greenhouse Gas emissions because of the fungicide sprays that are required to treat the disease.

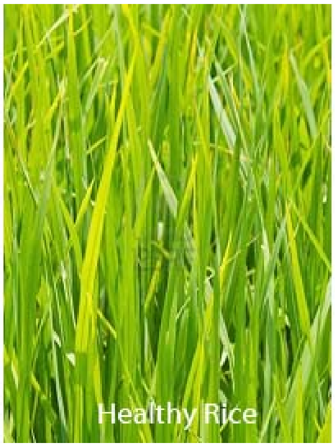

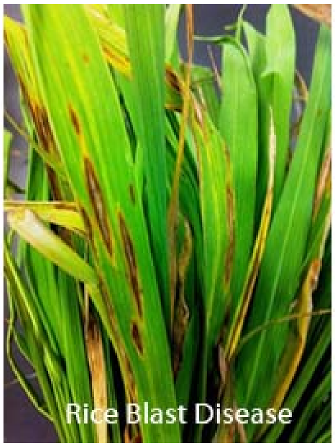

In the next questions we want you to indicate your preferences between two rice varieties at the indicated prices:

(1) Variety A that would not require fungicide applications
(2) a Conventionally-bred rice variety

Q8.2 *(The same choices are repeated with decreasing prices for Conventional variety until Conventional variety is chosen: € 20, € 15, € 10, € 8, € 5, € 3, € 2.25, € 2, € 1, € 0.5)*
Assume that you are buying non fragrant long grain white rice and need a 2.25 kg bag. Indicate your preferences between two rice varieties at the indicated prices:

(1) Variety A that would not require fungicide applications
(2) a Conventionally-bred rice variety

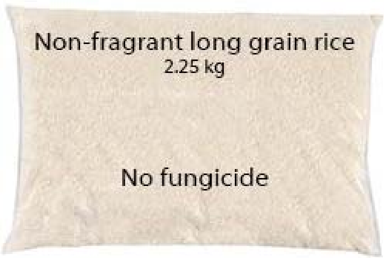

Variety A
No Fungicide
€2.25

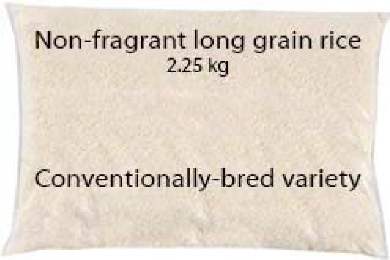

Conventional variety
€50.00

Consumption And Purchasing Practices

Q9.1
Had you heard of Cisgenic breeding prior to receiving this survey? Yes / No

Q9.2
Do you eat rice? Yes / No

Q9.3
In the last 14 days how many times did you eat rice? 0 / 1 / 2 / 3 / 4 / 5 / 6 / 7 / 8 / 9 / 1 0 / 11 / 12 13 / 14 / More than 14

Q9.4
Do you purchase any of the groceries for your household? Yes / No

Q9.5 *(Asked only if "Yes" to 9.4):*
How important are the following when you purchase groceries?

	Not at all Important	Slightly Important	Moderately Important	Very Important	Extremely Important
Price					
Brand Name					
Origin of Production					
Organic Status					
Nutritional Content					
Taste					
Product Quality					

Q9.6
What percent of your household food purchases are organic?

	0%	1-24%	25-49%	50-74%	75-99	100%	Not applicable
Fruits, Vegetables and	●	●	●	●	●	●	●
Meat	●	●	●	●	●	●	●
Dairy	●	●	●	●	●	●	●

Q9.7
Would you be willing to consume a Genetically Modified (GM) food if it were available?
Yes / No / Not enough information to decide

Q9.7.a ***(Asked only if “No” to Q9.7)***
How important are the following factors in your decision **not** to consume a Genetically Modified (GM) food product?

	Not at all Important	Slightly Important	Moderately Important	Very Important	Extremely Important
Effects on the environment					
Effects on health					
Ethical debate around genetically					

Q9.8: Would you be willing to consume a Cisgenic food if it were available?
Yes / No / Not enough information to decide

Q9.8.a ***(Asked only if “No” to Q9.8)***
How important are the following factors in your decision **not** to consume a Cisgenic food product?

	Not at all Important	Slightly Important	Moderately Important	Very Important	Extremely Important
Effects on the environment					
Effects on health					
Ethical debate around Cisgenic foods					

Q9.9: Have you ever knowingly eaten a Genetically Modified (GM) food product? Yes / No

Opinions About GM

Q10.1
We would like your opinion about **Genetically Modified (GM) rice**. Indicate the degree to which you agree or disagree with the following statements.

	Totally Disagree	Tend to Disagree	Tend to Agree	Totally Agree	Don't Know
GM rice is good for the national economy					
GM rice helps people in developing countries					
GM rice is safe for future generations					
GM rice is fundamentally unnatural					

GM rice is safe for my health and my family's health	
GM rice does no harm to the environment	
The development of GM rice should be encouraged	
I am in favor of allowing GM rice to be sold in my country	

Opinions About Cisgenic and Transgenic Rice

Q11.1
Researchers have discovered new ways of controlling common diseases in rice – things like blast. There are two new ways of doing this, both of which use Genetic Modification (GM). Both mean that the rice could be grown without fungicides, which lessens the Greenhouse Gas emissions of production. Both methods mitigate yield losses from blast which can be over 50% and can threaten the food security of the roughly 3.5 billion people who depend on rice in their diets.

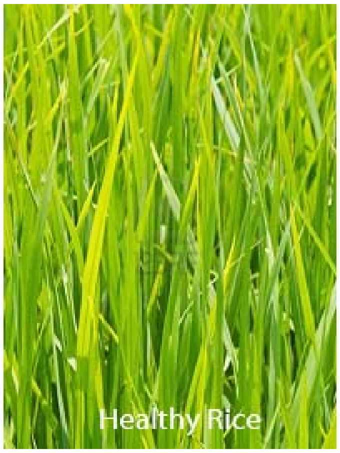

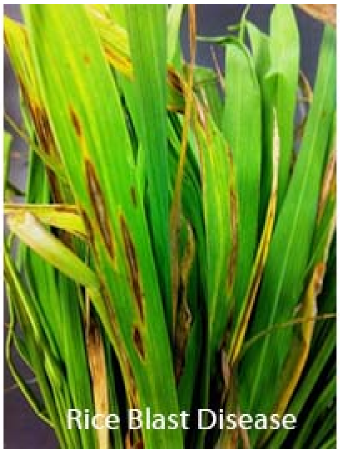

Q11.2
The first of the two methods is called **Cisgenic breeding**, which introduces a gene from a wild rice species into a commercial rice variety to make it resistant to rice blast disease.

Rice blast is a disease that requires fungicide sprays and decreases yields. Yield loss can be over 50% and can threaten the food security of the roughly 3.5 billion people who depend on rice in their diets.
For each of the following statements about this new technique please tell me if you agree or disagree.

	Totally	Tend to	Tend to	Totally	Don't

	Disagree	Disagree	Agree	Agree	Know
Cisgenic rice is good for the national economy					
Cisgenic rice helps people in developing countries					
Cisgenic rice is safe for future generations					
Cisgenic rice is fundamentally unnatural					
Cisgenic rice is safe for my health and my family's health					
Cisgenic rice does no harm to the environment					
The development of Cisgenic rice should be encouraged					
I am in favor of allowing Cisgenic rice to be sold in my country					

Q11.3
Which of the following statements is closest to your view?

- Rice created by Cisgenic breeding is Genetically Modified (GM) rice and should be clearly identified with a special label.
- Rice created by Cisgenic breeding is the same as ordinary rice and would not need special labeling.

Q11.4
The second method is called **Transgenic breeding**, which introduces a gene from another species into a commercial rice variety to make it resistant to rice blast disease.

Rice blast is a disease that requires fungicide sprays and decreases yields. Yield loss can be over 50% and can threaten the food security of the roughly 3.5 billion people who depend on rice in their diets.
For each of the following statements about this new technique please tell me if you agree or disagree.

	Totally Disagree	Tend to Disagree	Tend to Agree	Totally Agree	Don't Know
Transgenic rice is good for the national economy					
Transgenic rice helps people in developing countries					
Transgenic rice is safe for future generations					
Transgenic rice is fundamentally unnatural					
Transgenic rice is safe for my health and my					

family's health	
Transgenic rice does no harm to the environment	
The development of Transgenic rice should be encouraged	
I am in favor of allowing Transgenic rice to be sold in my country	

Q11.5
Which of the following statements is closest to your view?

- Rice created by Transgenic breeding is Genetically Modified (GM) rice and should be clearly identified with a special label.
- Rice created by Transgenic breeding is the same as ordinary rice and would not need special labeling.

Demographics
Q12.1
In what country do you currently live?

- Belgium
- France
- The Netherlands
- Spain
- The United Kingdom
- Other Country

Q12.2
How would you describe your living environment? Very rural / Somewhat rural / Suburban / Somewhat urban / Very urban

Q12.3
What is your age?

Q12.4
What is your gender? Male / Female

Q12.5: Do you live alone or with others? Live alone / Live with others

Q12.6 ***(asked only if "Live with others" in Q12.5)***

How many people in your household are in the following age categories?

Adults and children age 15 and older	
Children age 7 to 14 years old	
Children age 0 to 6 years old	

Q12.7

What is your highest education level?

- Primary school diploma
- General Certificate of Secondary Education
- Sixth Form
- Undergraduate at university
- Master's degree at university
- Doctoral degree (PhD)
- Higher education (not university)

Q12.8

What is your total net (after tax) household income?

- Less than 18 000 €
- 18 000 € to 34 999 €
- 35 000 € to 49 999 €
- 50 000 € to 64 999 €
- 65 000 € to 79 999 €
- 80 000 € to 99 999 €
- 100 000 or more €
